# Nano Ultrasound Contrast Agent for Synergistic Chemo‐photothermal Therapy and Enhanced Immunotherapy Against Liver Cancer and Metastasis

**DOI:** 10.1002/advs.202300878

**Published:** 2023-05-10

**Authors:** Yijie Qiu, Zhihua Wu, Yanling Chen, Jinghan Liao, Qi Zhang, Quan Wang, Yi Duan, Ke Gong, Sheng Chen, Liting Wang, Peili Fan, Yourong Duan, Wenping Wang, Yi Dong

**Affiliations:** ^1^ Department of Ultrasound Zhongshan Hospital Fudan University 180th Fenglin Road Shanghai 200032 China; ^2^ State Key Laboratory of Oncogenes and Related Genes Shanghai Cancer Institute Renji Hospital School of Medicine Shanghai Jiao Tong University 2200/25 Xietu Rd Shanghai 200032 China; ^3^ Department of Ultrasound Xinhua Hospital Affiliated to Shanghai Jiaotong University School of Medicine 1665 Kongjiang Road Shanghai 200092 China

**Keywords:** arsenic trioxide, ferroptosis, immunotherapy, nano ultrasound contrast agent, photothermal therapy

## Abstract

Advanced liver cancer is the most fatal malignant cancer, and the clinical outcomes of treatment are not very satisfactory due to the complexity and heterogeneity of the tumor. Combination therapy can efficiently enhance tumor treatment by stimulating multiple pathways and regulating the tumor immune microenvironment. Nanodrug delivery systems have become attractive candidates for combined strategies for liver cancer treatment. This study reports a nano ultrasound contrast agent (arsenic trioxide (ATO)/PFH NPs@Au‐cRGD) to integrate diagnosis and treatment for efficient ultrasound imaging and liver cancer therapy. This nanodrug delivery system promotes tumor‐associated antigens release through ATO‐induced ferroptosis and photothermal‐induced immunogenic cell death, enhancing the synergistic effects of ATO and photothermal therapy in human Huh7 and mouse Hepa1–6 cells. This drug delivery system successfully activates the antitumor immune response and promotes macrophage M1 polarization in tumor microenvironment with low side effects in subcutaneous and orthotopic liver cancer. Furthermore, tumor metastasis is inhibited and long‐term immunological memory is also established in orthotopic liver cancer when the nanodrug delivery system is combined with anti‐programmed death‐ligand 1 (PD‐L1) immunotherapy. This safe nanodrug delivery system can enhance antitumor therapy, inhibit lung metastasis, and achieve visual assessment of therapeutic efficacy, providing substantial potential in clinic applications for liver cancer.

## Graphical abstract

Mechanistically, ATO‐induced ferroptosis and photothermal‐induced immunogenic cell death enhance synergistic anti‐tumor effects and immune response in liver cancer. Liver tumor metastasis is inhibited and anti‐tumor immunity is stimulated when combined with anti‐programmed cell death‐ligand 1 (PD‐L1) immunotherapy. This work provides experimental evidence for using a nano ultrasound contrast agent to combine immunotherapy and chemo‐photothermal therapy against liver cancer.

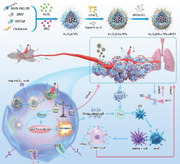

## 1 Introduction

Liver cancer remains a major contributor to the worldwide cancer burden with an estimated 1 million patients annually by 2025.^[^ ^1^ ^]^ Most patients are diagnosed at an advanced stage and do not meet the indications for surgical treatment.^[^ ^2^, ^3^ ^]^ In this case, traditional treatments, including thermal ablation, chemotherapy, and immunotherapy, remain unsatisfactory due to the ineffectiveness of a single modality or to the complexity and heterogeneity of tumor.^[^ ^4^, ^5^, ^6^ ^]^ The metastasis of liver cancer presents another difficulty in treatment and seriously affects the prognosis. Therefore, research on effective combination therapy to improve treatment efficacy and reduce metastasis is essential.^[^ ^7^, ^8^ ^]^ Combined chemo‐photothermal therapy (PTT) exhibits a better anticancer effect by utilizing the synergistic effects of dual therapy.^[^ ^9^, ^10^ ^]^ PTT is an anti‐cancer strategy that precisely targets tumors with minimal damage to surrounding healthy tissues.^[^ ^11^ ^]^ Gold nanoparticles (AuNPs) have been applied for PTT due to their good biocompatibility and excellent optical properties.^[^ ^12^, ^13^ ^]^ The photothermal effect of AuNPs can damage tumor tissues or release therapeutic molecules, such as tumor‐associated antigens (TAAs).^[^ ^14^ ^]^ Nevertheless, due to the limited therapeutic effect of AuNPs alone, their application is limited. The synergistic effect of combined chemo‐PTT may be a promising strategy for improving PTT efficacy.^[^ ^10^, ^15^ ^]^

Arsenic trioxide (ATO) exhibits a significant clinical benefit for the palliative treatment of unresectable primary liver cancer.^[^ ^16^ ^]^ However, the high systemic toxicity, rapid renal elimination of ATO, and low delivery efficiency to liver cancer greatly reduce the therapeutic efficacy and lead to severe systemic toxicity.^[^ ^17^, ^18^, ^19^ ^]^ Therefore, the clinical application of ATO in liver tumors is limited. Based on the above, we envisage that the use of nanotechnology for nanodrug delivery can significantly improve the efficacy of ATO, reduce toxicity, and achieve targeted drug delivery in liver cancer.^[^ ^19^, ^20^ ^]^ Since ATO has a strong affinity for sulfhydryl group (SH) in proteins, it can theoretically inactivate a variety of enzymes containing SH, such as glutathione (GSH).^[^ ^21^, ^22^ ^]^ GSH could assist glutathione peroxidase 4 (GPX4) in reducing toxic lipid peroxides (LPOs) to nontoxic hydroxy compound (LOH) to prevent LPO‐mediated damage to cell membrane structure and function.^[^ ^23^, ^24^ ^]^ The depletion of GSH could result in the inhibition of GPX4 activity and a corresponding increase in intracellular LPO levels, ultimately leading to ferroptosis.^[^ ^23^, ^25^, ^26^ ^]^ Therefore, ATO may induce ferroptosis through GSH depletion and GPX4 inactivation. Moreover, the ferroptosis‐induced release of TAAs from dying cancer cells could further enhance the immune response.

Immunotherapy has made significant progress in liver cancer treatment and has been found to be a promising strategy for patients with advanced liver cancer.^[^ ^27^, ^28^ ^]^ Recently, anti‐programmed cell death‐legand 1(PD‐L1) mAb was approved by the U.S. Food and Drug Administration (FDA) for the treatment of several cancers.^[^ ^29^, ^30^ ^]^ It restores the functions of T cells in tumor microenvironment (TME) by preventing interactions between programmed death 1(PD‐1) and PD‐L1.^[^ ^31^ ^]^ However, anti‐PD‐L1 mAb is effective in only a small number of patients and achieves a clinical remission rate of ≈15−20%,^[^ ^2^, ^32^ ^]^ possibly due to inadequate TAAs release and the highly immunosuppressive nature of TME.^[^ ^33^ ^]^ Therefore, promoting TAAs release by combined chemo‐PTT to improve the anti‐PD‐L1 mAb therapeutic response may be an approach for successful liver cancer treatment.

**Figure 1:**
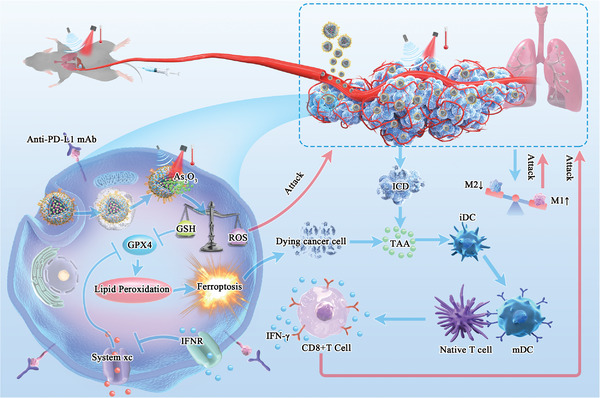
A schematic diagram of ATO /PFH NPs@Au‐cRGD assisted by ultrasound and photothermal for liver cancer and metastasis. ATO induces ferroptosis of tumor cells through GSH depletion and increased GPX4; and the increased ROS can also directly kill tumor cells, thereby enhancing the synergistic effect. Ferroptosis and photothermal effects further promote the transformation of immature DC into mature DC and macrophage M1 polarization in TME, and then activate the native T cells to become cytotoxic T lymphocyte, thus inhibiting the tumor growth. The anti‐PD‐L1 mAb further promotes the anti‐tumor effect of cytotoxic T lymphocyte by blocking the PD‐1 / PD‐L1 axis which enable synergistic treatment of orthotopic liver cancer, and enhance the immune response, thereby reduce lung metastasis.

Furthermore, ultrasound‐targeted microbubble destruction (UTMD) can be exploited to change the structures of cells to facilitate the targeted release of nanodrugs into tumor cells which could improve the efficacy of tumor treatment and prevent the systemic toxicity of antitumor drugs.^[^ ^34^, ^35^, ^36^ ^]^ The nanoparticles (NPs) accumulate in tumor tissues through the enhanced permeability and retention effect (EPR) and targeting effect. Then, UTMD can increase the permeability of the cell membrane through the cavitation effect so that the drugs loaded in NPs are more easily released and taken up.^[^ ^36^ ^]^ Moreover, NPs can also load imaging agents for visualizing therapy, such as perfluorohexane (PFH). Under these circumstances, we constructed a nanodrug delivery system based on the nano ultrasound contrast agent of ATO/PFH NPs@Au‐cRGD for the codelivery of ATO, and PFH, conjugated with AuNPs and the liver cancer‐specific targeting peptide cRGD‐SH (**Figure** **1**). Since *α*v*β*3 integrin‐specific ligands are overexpressed in liver cancer and neovascularization cells, a cyclic arginylglycylaspartic acid peptide (cRGD) can be bound to AuNPs via gold–sulfur bonds for targeted liver cancer therapy.^[^ ^37^ ^]^ The AuNPs were deposited onto liposome surfaces, followed by the formation of gold nanoshells. NPs combined with cRGD‐SH and UTMD can achieve better targeted delivery. As a stable, safe, and nontoxic liquid, PFH can undergo a phase transition and enhance the ultrasound echo signal under ultrasonic stimulation and photothermal effects. This nano ultrasound contrast agent could induce tumor ferroptosis through ATO and antagonize tumors through PTT, which will lead to increased antigens release, promote the maturation of dendritic cells (DCs), trigger an adaptive T‐cell response, improve immune activity, and further enhance the efficacy of anti‐PD‐L1 mAb therapy. Briefly, this nanodrug delivery system could realize visual imaging, enable synergistic treatment of liver cancer, and enhance the immune response and influence the phenotypes of macrophages in TME, thereby achieving enhanced liver tumor therapy, reducing metastasis, and enabling visual assessment of therapeutic efficacy.

## 2 Results and Discussion

### 2.1 Synthesis, Characterization, and Imaging Effect of ATO/PFH NPs@Au‐cRGD

**Figure 2:**
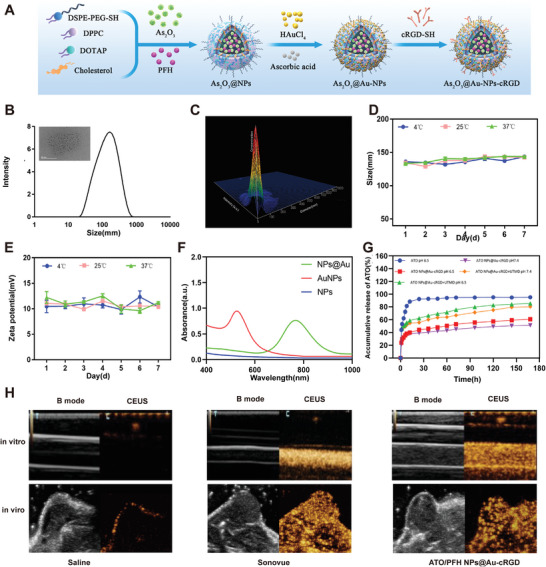
Physicochemical characterization, in vitro and in vivo ultrasound imaging efficacy, and photothermal ability of NPs). A) Schematic illustration of ATO/PFH NPs@Au‐cRGD. B) Size distribution and TEM images of ATO/PFH NPs@Au‐cRGD. Scale bar = 50 nm. C) Concentration distribution of NPs. D) and E) Storage stability of ATO/PFH NPs@Au‐cRGD. F) UV–vis spectra of liposome NPs, and liposome NPs@Au. G) In vitro release profiles of ATO with or without UTMD treatment at pH 6.5 and pH 7.4. (H) Ultrasound imaging efficacy of NPs i*n vitro* and in vivo compared with saline or Sonovue. The data are presented as means ± SD.

The filming‐rehydration method was utilized to prepare and synthesize ATO/PFH NPs@Au‐cRGD according to previously reported method with minor modifications (**Figure** **2**A).^[^ ^38^ ^]^ DSPE‐PEG_2K_ is a biocompatible, biodegradable, and amphiphilic material that can prolong blood circulation and improve stability and encapsulation efficiency.^[^ ^39^ ^]^ Therefore, DSPE‐PEG_2K_ was chosen as a backbone to construct aptamer‐functionalized core‐shell nanoparticles with ATO and PFH encased in the core and AuNPs as the shell. DSPE‐PEG_2k_‐SH, DOTAP, DPPC, and cholesterol formed lipid membranes to load ATO and PFH, and to synthesize ATO/PFH NPs. The ATO/PFH NPs @Au were developed by the deposition of AuNPs onto the surface of liposomes via reduction of chloroauric acid (HAuCl_4_) following a previously reported method.^[^ ^40^ ^]^ Then, RGD‐SH binds to the gold shell through SH bonds. Transmission electron microscopy (TEM) confirmed the deposition of AuNPs on the liposome surface (Figure 2A). The hydrodynamic diameter was 121 ± 5 nm and Zeta potential was 10.47 ± 1.20 mV in aqueous solution (Figure 2B and Figure S1, Supporting Information). Concentration analysis showed that the size range of the ATO/PFH NPs@Au‐cRGD was ≈100−150 nm (Figure 2C). After storage at 4, 25, and 37 °C in phosphate‐buffered saline (PBS) and serum (5%, pH 7.4) for 7 days, there was no obvious change in particle size and Zeta potential, indicating good stability of the synthesized nanoparticles (Figure 2D,E). The results of UV–vis spectra showed that ATO/PFH NPs@Au‐cRGD had characteristic plasmonic coupling (between the AuNPs) absorption peak at 760 nm, while no resonance peak at the near‐infrared wavelengths was detected in non‐coated liposomes (Figure 2F). AuNPs formed a gold shell surrounding the liposome core, which was also confirmed by energy‐dispersive X‐ray spectroscopy (EDS) analysis (Figure S2, Supporting Information).

To evaluate the controlled release ability of our nanodrug delivery system, the drug release characteristics of ATO were detected at pH 7.4 and 6.5 (pH 6.5 was used to simulate the slightly acidic TME). The drug release profiles are shown in Figure 2G. Compared to the fast release of free ATO (≈92% in 24 h), ATO NPs‐cRGD showed significant sustained release. Specifically, the release rates of ATO from ATO NPs‐cRGD with UTMD at pH 6.5 were as high as 85.3% ± 0.4% at 7 days, but ≈62.4% ± 0.4% at 12 h. What's more, ATO can be effectively released with the aid of an ultrasound therapeutic apparatus and low acidity TME (pH 6.5). When combined with UTMD, the release rate of ATO in the low acidity TME (7 days) was 85.3% ± 0.4%, while that without UTMD was 60.8% ± 0.5%. The statistical analysis showed that the release efficiency of ATO combined with UTMD was significantly high than that of without UTMD (*p* < 0.001) (Figure S3). The acoustic cavitation effect generated by UTMD is a process of vibration, expansion, contraction, and rupture of microbubbles, which lead to the destruction of the nanocarrier and the release of the drug encapsulated in the nanoparticles, thus promoting the selective release at tumor site.^[^ ^41^, ^42^, ^43^ ^]^

To evaluate the ultrasound imaging ability of ATO/PFH NPs@Au‐cRGD, in vivo and in vitro imaging experiments were carried out with saline as a negative control and SonoVue contrast agent as a positive control. As shown in Figure 2H, the contrast‐enhanced images of SonoVue and ATO/PFH NPs@Au‐cRGD were significantly enhanced in vitro after ultrasound exposure. The in vivo ultrasound imaging efficacy of the nano‐contrast agent was determined, and similar results were detected (Figure 2H). Due to its high boiling point and stability against passive vaporization in physiological conditions, PFH is usually selected as the appropriate perfluoropentane for ultrasound contrast‐agents in a nanodroplet form.^[^ ^44^, ^45^, ^46^ ^]^ However, it needs higher energy to trigger phase‐transition for ultrasound imaging due to its higher boing point, which may have adverse effect on UTMD. Compared to commercial ultrasound contrast agents, the ATO/PFH NPs@Au‐cRGD exhibited similar ultrasound imaging effects in tumors, clearly showing contrast enhancement in contrast mode, which could assess the dynamic blood perfusion of tumors visually thereby realizing precise molecular imaging during treatment.

To investigate the photothermal properties of the ATO/PFH NPs@Au‐cRGD, a 760 nm laser was used to irradiate PBS, AuNPs, ATO, and ATO NPs@Au‐cRGD (Figure S4, Supporting Information). Representative thermal images of PBS, AuNPs, ATO, ATO NPs@Au‐cRGD (under NIR irradiation (1.0 W cm^−2^) and the changes in temperature with radiation time were recorded (Figure S4A,B, Supporting Information). After 4 min of irradiation, the maximum temperatures of the AuNPs and ATO NPs@Au‐cRGD were 54.2 ± 0.6 °C and 48.7 ± 0.7 °C respectively. This slight difference may be due to the difference in refractive index of the surrounding media, and thermal conductivity. It's reported that the refractive index of the surrounding media in AuNPs is 1.33 (similar to water), however, the refractive index of PEG‐modified AuNPs is 1.57.^[^ ^47^ ^]^ In addition, the thermal conductivity of PEG, and water are also different, which are 0.31, and 0.6 W m^−1^ K^−1^, respectively. What's more, previous studies have shown that PEG‐modified AuNPs demonstrate lower thermal conductivity than unmodified AuNPs.^[^ ^48^ ^]^ The difference in the refractive index and thermal conductivity will lead to the difference in photothermal properties. However, ATO NPs@Au‐cRGD still exhibits stable and high photothermal performance for photothermal therapy. The temperature of PBS and ATO was relatively unchanged after irradiation (22.1 ± 0.3 °C vs 22.0 ± 0.2 °C). Moreover, the nanoparticles had excellent photothermal stability with no difference in temperature changes across five irradiation and cooling cycles (Figure S4C, Supporting Information).

### 2.2 Cell uptake and cytotoxicity of ATO/PFH NPs@Au‐cRGD

**Figure 3:**
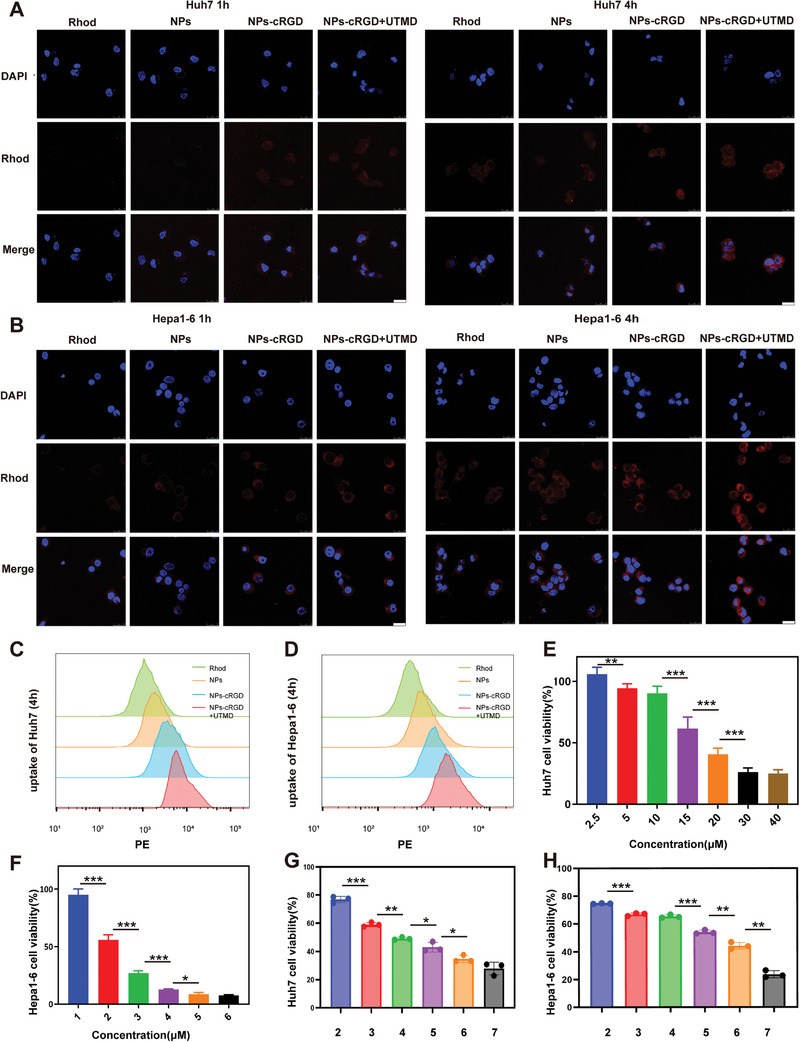
The cell uptake and cell cytotoxicity of ATO/PFH NPs@Au‐cRGD. A) CLSM of the Huh7 intracellular uptake in Rhod, Rhod NPs@Au, Rhod NPs@Au‐cRGD, Rhod NPs@Au‐cRGD +UTMD at hour 1 and hour 4. Scale bar = 25 µm. B) CLSM and quantification analysis of the Hepa1‐6 intracellular uptake in Rhod, Rhod NPs@Au, Rhod NPs@Au‐cRGD, Rhod NPs@Au‐cRGD +UTMD at hour 1 and hour 4. Scale bar = 25 µm. Flow cytometry analysis of intracellular uptake in Huh7 cells (C) and Hepa1‐6 cells (D) in different groups. Cell viability of Huh7 (E) and Hepa1‐6 cells (F) in different concentrations. Cell viability of Huh7 (G) and Hepa1‐6 cells (H) in different groups. 2 AuNPs,3 ATO,4 ATO NPs@Au,5 ATO NPs@Au‐cRGD,6 ATO NPs@Au‐cRGD +UTMD,7 ATO NPs@Au‐cRGD+UTMD+laser. All data are presented as the mean ± SD. *p < 0.05, **p < 0.01, ***p < 0.001.

An effective cellular uptake ability of drugs is a prerequisite for a good antitumor effect so the uptake ability of nanoparticles by Huh7 and Hepa1‐6 cells was evaluated. As shown in **Figure** **3**A,B, the fluorescence intensity detected by confocal laser scanning microscopy (CLSM) was free Rhod < Rhod NPs@Au < Rhod NPs@Au‐cRGD < Rhod NPs@Au‐cRGD+UTMD in the Huh7 and Hepa1‐6 cell lines. The measurement results of flow cytometry in Huh7 and Hepa1‐6 cells (Figure 3C,D) were consistent with CLSM. The Rhod NPs‐cRGD+UTMD group had the highest intracellular fluorescence intensity, indicating that due to the modification of cRGD‐SH and UTMD, the absorption of nanoparticles was significantly increased. The IC_50_ concentrations of ATO in Huh7 and Hepa1‐6 cells were 15.71 and 2.23 µm respectively (Figure 3E,F). In addition, 2 W cm^−2^ of 1 MHz UTMD for 1 min and 1 W cm^−2^ of 760 nm NIR laser irradiation for 4 min had little effect on Huh7 cells (Figures S5 and S6, Supporting Information); therefore, it was used in subsequent experiments. To assess the effects of ATO and other treatments on Huh‐7 and Hepa1‐6 cells, CCK8 experiments were conducted. After 24 h of treatment, compared with the free ATO and other nanoparticle groups, the cell activity of the ATO/PFH NPs@Au‐cRGD+UTMD+laser group was significantly reduced, and the antiproliferative effect was the strongest under the action of UTMD and laser (*p* < 0.05; Figure 3G,H).

### 2.3 ATO NPs@Au‐cRGD+UTMD+Laser Exerted Superior Antitumor Effects In Vitro By Inducing ROS Production and GSH Depletion

**Figure 4:**
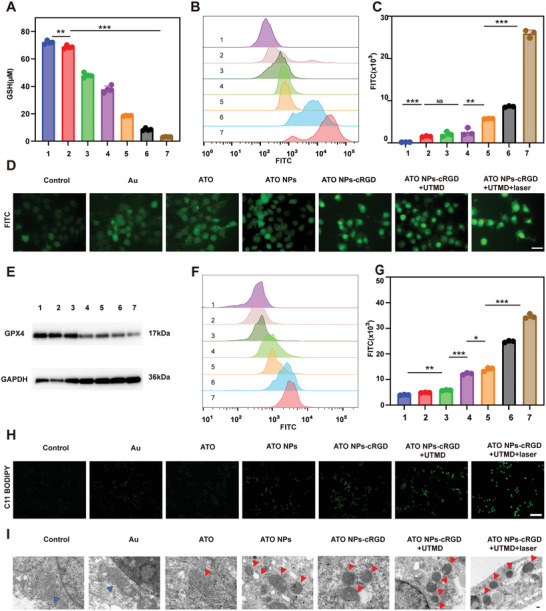
Mechanism of ferroptosis induced by ATO/PFH NPs@Au‐cRGD. A) GSH levels in Huh‐7 cells treated with different interventions. B) Flow cytometry analysis of ROS in Huh7 cells with different treatments. C) Quantification of flow cytometry analysis of Huh7 cells with different treatments. D) Fluorescence images of ROS in Huh7 cells. Scale bar = 50 µm. E) Western blotting results of GPX4 expression in Huh‐7 cells after different treatments. F) Flow cytometry analysis and G) quantitative fluorescence intensity of LPOs in Huh7 cells with different treatments. H) Fluorescence images of LPOs in Huh7 cells. The fluorescence of C11 BODIPY channel represents the level of LPOs. Scale bar = 100 µm. I) Bio‐TEM images of Huh7 cells after different treatments for 24 h. Red triangle marks indicate mitochondria damage of Huh7 cells, while blue triangle marks indicate healthy mitochondria of Huh7 cells. Scale bars = 100 nm. (1, PBS; 2, AuNPs 3, ATO; 4, ATO NPs@Au; 5, ATO NPs@Au‐cRGD; 6, ATO NPs@Au‐cRGD+UTMD; 7, ATO NPs@Au‐cRGD+UTMD+laser). All data are presented as the mean ± SD, *p < 0.05, **p < 0.01, ***p < 0.001.

After treatment with different formulations, the GSH levels in the Huh7 and Hepa1‐6 cell lines were assessed. Under the intervention of ATO and nanoparticles, GSH level decreased significantly, with the lowest GSH level in the ATO NPs@Au‐cRGD+UTMD+laser group, indicating that this nanodelivery system could effectively induce GSH depletion (**Figure** **4**A and Figure S7, Supporting Information). The reactive oxygen species (ROS) indicator 2′,7′‐dichlorodihydrofluorescein was introduced to detect ROS generation by measuring fluorescence intensity. After ATO NPs@Au‐cRGD+UTMD+laser treatment, ROS was the highest in this group, as measured by flow cytometry and fluorescence intensity quantitative analysis (*p* < 0.001) (Figure 4B,C and Figure S7B,C, Supporting Information). Immunofluorescence images indicated similar results (Figure 4D), which revealed that the presence of ATO induced increased ROS levels and GSH depletion.

Whether the ATO released from NPs can induce ferroptosis to obtain anti‐tumor effects was not clear, so relevant experiments were performed. The protein expression level of the ferroptosis‐related protein GPX4 was measured, and the ATO NPs@Au‐cRGD+UTMD and ATO NPs@Au‐cRGD+UTMD+laser groups showed significantly decreased GPX4 expression (Figure 4E and Figure S8, Supporting Information). It has been reported that the accumulation of intracellular LPOs eventually induces ferroptosis.^[^ ^49^, ^50^ ^]^ The CLSM images showed that C11 BODIPY fluorescence probe displayed the similar increase in LPOs (Figure 4H and Figure S9, Supporting Information). Flow cytometry showed that LPOs were increased significantly after treatment with ATO nanoparticles (Figure 4F,G and Figure S10, Supporting Information), which could cause ferroptosis in tumor cells. Bio‐TEM images of Huh7 and Hepa1‐6 cells after different treatments showed that this nanodelivery system could cause ferroptosis in Huh7 and Hepa1‐6 cells. Red triangle marks indicate vacuolated mitochondria of Huh7 and Hepa1‐6 cells after severe oxidative damage, while blue triangle marks indicate relatively healthy mitochondria (Figure 4I).

### 2.4 Biodistribution and Antitumor Ability of ATO NPs@Au‐cRGD+UTMD+Laser In Vivo

**Figure 5:**
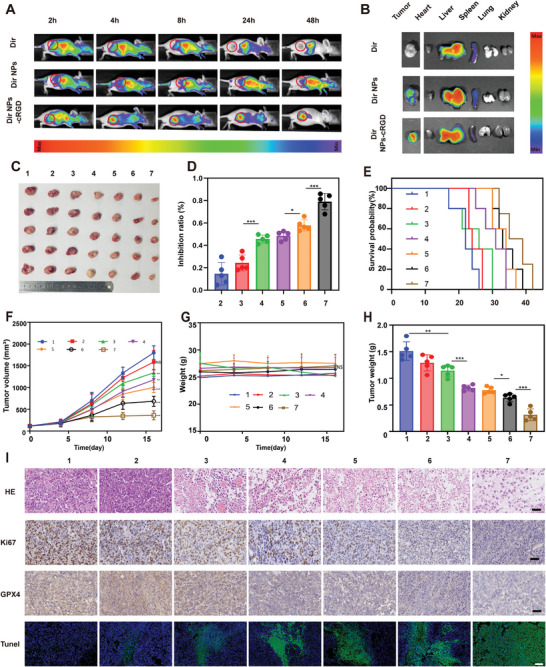
Tumor‐targeting and antitumor activity of ATO NPs@Au‐cRGD+UTMD+laser in mice bearing Huh7 tumor. A) Biodistribution of free Dir, Dir NPs, and Dir NPs‐cRGD in Huh7 tumor‐bearing mice. B) Fluorescence intensity of excised tumors and major organs of various groups. C) Representative images of tumor tissues in different groups. D) Tumor inhibition ratio of different treatments. E) Survival rate in different treatment groups. F) Tumor growth curve with time under various treatments. G) The weight of mice with time under various treatments. H) The tumor weight of various treatments. I) HE, Ki67, GPX4, and, TUNEL immunohistochemistry staining of excised tumors after treatment ended. Scale bar = 100 µm (1, PBS; 2, AuNPs 3, ATO; 4, ATO NPs@Au; 5, ATO NPs@Au‐cRGD; 6, ATO NPs@Au‐cRGD +UTMD; 7, ATO NPs@Au‐cRGD +UTMD+laser). All data are presented as the mean ± SD. *p < 0.05, **p < 0.01, ***p < 0.001.

Accurate enrichment of ATO at tumor sites is important for improving therapeutic efficacy and reducing side effects on other normal organs. To confirm the tumor‐targeting role of NPs, the biodistribution of NPs in mice was assessed by in vivo imaging. Free Dir, Dir NPs, and Dir NPs‐RGD were injected into mice through the tail vein when the tumor volume reached 200 mm^3^. After injection, the fluorescence distribution in vivo was observed using the small animal in vivo imaging system at 2, 4, 8, 24, and 48 h (**Figure** **5**A). Compared with the Dir and Dir NP groups, the Dir NPs‐RGD group had higher accumulated bioluminescence in the tumor at 48 h, implying that the RGD‐NPs had better tumor‐targeting ability. Each group of mice was sacrificed after 48 h, the tumors and major organs of mice were removed, and ex vivo imaging was performed. The fluorescence intensity of the tumors in the Dir NP‐RGD group was the strongest (Figure 5B). The NPs‐cRGD group had significantly higher drug accumulation in tumors than in organs. In brief, these differences indicated that the ATO NPs@Au‐cRGD group had a superior tumor‐targeting ability. Temperature changes were also recorded using an infrared thermal imager (Figure S11, Supporting Information). In the ATO NPs@Au‐cRGD+UTMD+laser group, the temperatures of the tumor sites increased significantly, reaching 49 °C within 4 min after irradiation (Figure S11, Supporting Information). These data demonstrated that RGD and AuNP‐ modified nanodrug delivery systems had in vivo photothermal effects.

The therapeutic effects of various treatments in vivo were further examined. When the tumor volume of each mouse reached ≈100 mm^3^, the mice were divided into seven groups, and different formulations were injected through the tail vein every 3 days (with/without UTMD and laser) according to the flowchart (Figure S12, Supporting Information, *n* = 5). Laser irradiation was applied to the laser‐treated groups 12 h post‐injection. After the different treatments, the tumor volume of the mice was observed to be in the sequence PBS> Au> ATO> ATO NPs> ATO NPs‐cRGD> ATO NPs‐cRGD+UTMD>ATO NPs‐cRGD+UTMD+laser (Figure 5C,F). On day 15, the tumor size in the ATO NPs@Au‐cRGD + UTMD +laser group was significantly reduced, demonstrating outstanding antitumor ability compared with other groups. On day 16, we euthanized each mouse and harvested vital organs or tumors for further experiments. First, the weight of tumors removed from the mice showed that the average tumor weight of the mice in the PBS group reached 1.5 g, which was ≈1.32 times that in the free ATO group, and 4.78 times that in the ATO NPs@Au‐cRGD + UTMD +laser group (Figure 5H). The ATO NPs@Au‐cRGD + UTMD+ laser group showed the best antitumor effect, with an inhibition rate of 79.1% (Figure 5D), and a relatively stable body weight, implying that this nanodrug delivery could effectively inhibit tumor growth, and reduce toxicity, and side effects. In order to study the pharmacokinetic properties of ATO, the dose of ATO in blood was measured at different time points. Compared to the ATO group, controlled release of ATO NPs@Au and ATO NPs@Au‐cRGD were evidenced by significantly prolonged half‐life, reduced clearance, and improved AUC (Table S1, Supporting Information). The improved pharmacokinetic behavior of ATO by liposome nanoparticles may result in enhanced drug accumulation at the tumor site. To precisely assess the distribution of drug delivered to main organs and tumor sites, the ATO concentration was determined 24 h after the administration. The ATO NPs @ Au‐cRGD had higher drug accumulation at tumor sites versus ATO group, indicating that the ATO NPs @ Au‐cRGD group has a superior tumor‐targeting ability (Figure S13, Supporting Information). In addition, the survival curves showed that all mice in the PBS group died before day 26, while the ATO NPs@Au‐cRGD+UTMD+laser group had the longest survival time (Figure 5E). The results of tumor HE staining showed that the area of tumor cell necrosis in the nanodrug delivery group was significantly higher than that in PBS group, indicating that the nanodrug delivery treatment group could cause tumor cell death (Figure 5I). The results of terminal deoxynucleotidyl transferase dUTP nick‐end labeling (TUNEL) experiments demonstrated that ATO NPs@Au‐cRGD+UTMD+laser had the strongest green fluorescence and the largest amount of apoptosis, indicating that this group had the best antitumor effect. Moreover, immunohistochemical analysis of Ki67 showed that tumor cell proliferation was significantly reduced in the ATO NPs@Au‐cRGD + UTMD +laser group, thereby inhibiting tumor cell proliferation. Notably, the GPX4 levels were also significantly decreased in the ATO NPs@Au‐cRGD+UTMD +laser groups, which induced ferroptosis in tumor cells, consistent with the results of western blotting analysis in the former cellular experiment section (Figure 4E).

The Response Evaluation Criteria in Solid Tumors (RECIST) provides a standardized set of tumor shrinkage‐based response evaluation rules that are globally available and interpretable by most clinicians.^[^ ^51^ ^]^ There are four types of antitumor efficacy evaluations: complete response (CR), partial response (PR), stable disease (SD), and progressive disease (PD). In our subcutaneous tumor models, the PBS group had 100% PD, and the disease control rate (consisting of CR, PR, and SD) was 0. For the AuNPs and ATO groups, the disease control rate was 25%, and the ATO group had a smaller tumor size. In the other nanoparticle groups, the disease control rates were all 50%, and the ATO NPs@Au‐cRGD+UTMD+laser group demonstrated the best antitumor effect. Taken together, these data suggested that this nanodrug delivery system had predominant active targeting properties and superior tumor killing effects.

### 2.5 The Immune Response Induced by ATO NPs@Au‐cRGD+UTMD+Laser In Vitro and In Vivo

**Figure 6:**
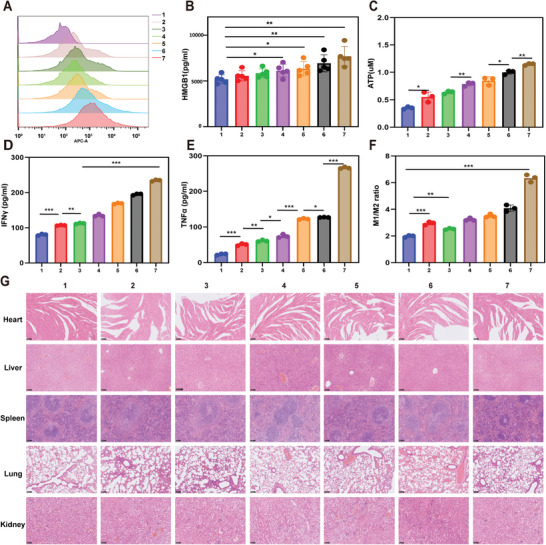
In vitro immunogenic cell death triggered by ATO NPs@Au‐cRGD+UTMD+laser and the immune response in vivo. A) Flow cytometry quantification of CRT of Huh7 cells. B) HMGB1 released from Huh7 cells detected by an enzyme‐linked immunosorbent assay (ELISA) kit. C) ATP secretion detected by an enhanced ATP assay kit. The ELISA analysis of the secretion of proinflammatory cytokines in tumor tissues of Balb/c, including IFN‐*γ* (D) and TNF‐*α* (E). F) The ratio of M1/M2 in different intervention. G) The HE staining result of important organs (heart, liver, spleen, lung, and kidney) from excised mice after treated with different formulations. Scale bar = 100 µm. (1, PBS; 2, AuNPs 3, ATO; 4, ATO NPs@Au; 5, ATO NPs@Au‐cRGD; 6, ATO NPs@Au‐cRGD +UTMD; 7, ATO NPs@Au‐cRGD +UTMD+laser). All data are presented as the mean ± SD. *p < 0.05, **p < 0.01, ***p < 0.001.

It was recently reported that immunogenic cell death (ICD) releases TAAs in early ferroptosis cells and PTT.^[^ ^52^ ^]^ The induced ICD was characterized by calreticulin (CRT) exposure, high mobility group box 1 (HMGB1) release, and adenosine triphosphate (ATP) secretion. As shown in **Figure** **6**A, CRT expression increased significantly, as detected by flow cytometry, after being incubation with different treatments for 24 h. HMGB1 release (Figure 6B and Figure S14, Supporting Information) and ATP secretion (Figure 6C and Figure S15, Supporting Information) were also greatly enhanced by the ATO NPs@Au‐cRGD+UTMD+laser as determined by enzyme‐linked immunosorbent assay (ELISA) and bioluminescence assay, respectively.

Tumor‐associated macrophages (TAMs) determine the efficacy of immunotherapy, which are generally classified into anti‐tumor M1 and pro‐tumor M2 phenotypes.^[^ ^53^ ^]^ CD86 and CD206, markers of M1 and M2 macrophages, were separately used to evaluate the ratio of M1/M2 macrophages in different treatment groups (Figure 6F). Flow cytometry analysis showed that the M1/M2 ratio in the ATO NPs@Au‐cRGD +UTMD (4.08), and ATO NPs@Au‐cRGD+UTMD+laser (6.33) groups was significantly higher than that in the PBS group (1.98), indicating that ATO NPs@Au‐cRGD +UTMD+laser could effectively promote the repolarization of M2 macrophages into M1 macrophages in TME. This is similar to the previous reports that nanogold‐mediated PTT treatment and ferroptosis can repolarize the TAMs from M2 to the M1 phenotype.^[^ ^54^, ^55^, ^56^ ^]^ In addition, the nanodelivery system significantly upregulated the levels of tumor necrosis factor‐*α* (TNF‐*α*), and interferon‐*γ* (IFN‐*γ*) in Huh7 liver tumors (Figure 6D,E), which are important indicators of tumor immunity. The ELISA results of TNF‐*α* and IFN‐*γ* were consistent with the above results of the flow analysis of immune cells, indicating inflammation in the tumor and improvement of tumor immunosuppression. In summary, the ATO NPs@Au‐cRGD+UTMD+laser group was confirmed to effectively remodel the immunosuppressive TME and promote the immune killing response. The HE images of the major organs showed no significant difference in the nanodrug delivery group and the PBS group, providing evidence that the nanodelivery system has excellent biocompatibility and biosafety (Figure 6G).

### 2.6 Effects of Combination Therapy on Immunological Response and Orthotopic Liver Tumor Growth and Metastasis

**Figure 7:**
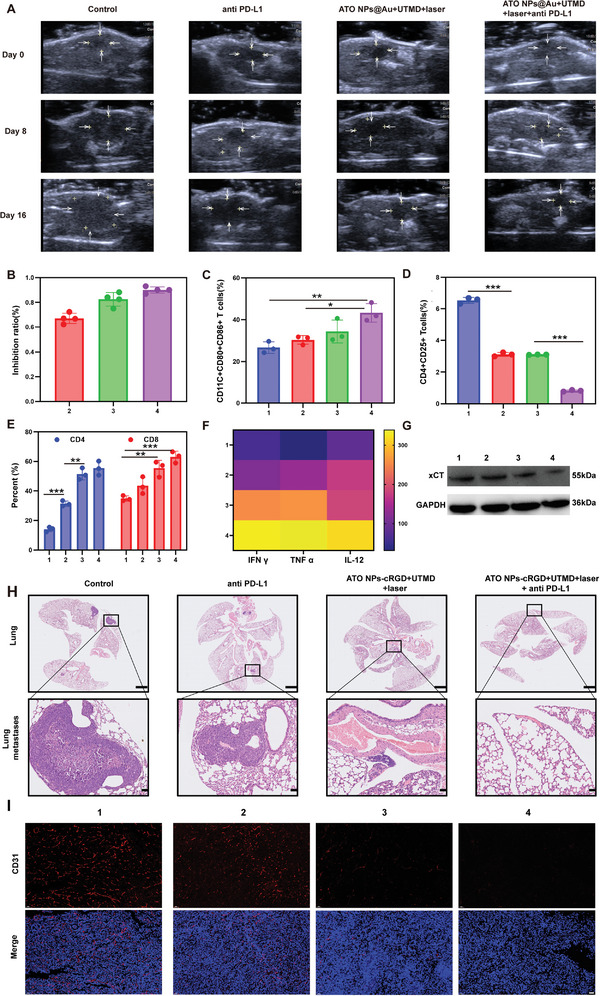
Antitumor and antimetastatic effects on orthotopic liver tumors. A) The ultrasound images of the C57 mice orthotopic liver tumors. B) The inhibition rate of the different treatments. Flow cytometric data of DC maturation (C) and Treg cells (D) in different groups. The percent of the CD4 and CD8 (E) in various treatments. (F) Cytokine levels in the mice tumor treated by different formulations. G) The protein xCT/GAPDH ratio in the tumor tissues after different treatments. (H) The HE staining of excised lung after various treatments ended. Scale bar = 1 mm for lung, 50 µm for lung metastases. (I) The CD31 analysis of mouse tumor by immunofluorescence staining. Scale bar = 50 µm. (1 PBS, 2 anti PD‐L1 mAb, 3 ATO NPs@Au‐cRGD+UTMD+laser, 4 ATO NPs@Au‐cRGD+UTMD+laser+anti PD‐L1 mAb). All data are presented as the mean ± SD, *p < 0.05, **p < 0.01, ***p < 0.001.

Tumor growth and metastasis are the main reasons for the failure of liver cancer treatment. Based on immune promotion triggered by ferroptosis and photothermal effects, we further explored whether our nanodrug delivery therapy combined with an anti‐PD L1 mAb could produce synergistic inhibition of in situ liver cancer and metastasis. The C57 mice were divided into four groups (*n* = 5 per group) and treated with PBS, anti‐PD‐L1 mAbs, ATO NPs@Au‐cRGD+UTMD+laser, and ATO NPs@Au‐cRGD+UTMD+laser+anti‐PD‐L1 mAbs (combination therapy). Anti‐PD‐L1 mAbs (100 µg per mouse) were injected 2, 5, 8, 11, and 14 d after irradiation (Figure S16, Supporting Information). The growth of liver cancer in situ was monitored by ultrasound imaging (**Figure** **7**A). The in situ liver cancer of the PBS group grew rapidly and the anti‐PD‐L1 mAb injection alone had a slight inhibitory effect on the growth of tumors. However, the in situ tumor inhibition rate of combination therapy reached 82.5% (Figure 7B), showing the best antitumor effect.

To examine the underlying mechanism of the synergistic antitumor effects of the ATO NPs@Au‐cRGD+UTMD+laser+anti‐PD‐L1 mAb treatment, the mice were sacrificed and the immune cells in situ tumors were analyzed by flow cytometry, ELISA, and western blot. As antigen‐presenting cells, DCs from mouse inguinal draining lymph nodes (LNs) were extracted for DCs maturation analysis. A significant increase in CD11c^+^CD80^+^CD86^+^ cells in mouse LNs of the combination therapy group (43.23% ± 4.43%) was detected, compared with that in the control group (26.63% ± 2.71%), in the anti‐PDL1 mAb group (30.3% ± 2.09%) and in the ATO NPs@Au‐cRGD+UTMD+laser group (34.3% ± 5.51%; Figure 7C and Figure S17, Supporting Information), which suggested that combination therapy can effectively stimulate DCs maturation to activate systemic immune activities. Moreover, the M1 cytokines TNF‐*α*, IFN‐*γ*, and IL‐12, which promote the killing effect of cytotoxic T lymphocytes (CTLs) were significantly higher in the combination therapy group than in the other three groups (Figure 7F). The IFN‐*γ* produced by T cells further decreased xCT protein secretion, thus enhancing the ferroptosis of tumor cells induced by our nanodrug delivery system (Figure 7G). Moreover, the percentages of tumor‐killing CD3^+^CD8^+^ CTLs (Figure S20, Supporting Information), immune‐activating CD3^+^CD4^+^ T helper cells (Th) (Figure S19, Supporting Information), and immunosuppressive CD4^+^CD25^+^ Treg cells (Figure S18, Supporting Information) were also evaluated and the results are shown in Figure 7D–E. The percentage of CTLs in the combination therapy group increased significantly (63.03% ± 3.84%) compared with that in the anti‐PD‐L1 mAb treatment group (43.47% ± 5.75%), which attributed to the increased release of TAAs to recruit CTLs to the tumor site. Compared to anti‐PD‐L1 mAb treatment alone, the percentage of Th cells (31.37% ± 1.84% vs 55.33% ± 4.75%) and DCs (30.3% ± 2.09% vs 43.23% ± 4.43%) also increased significantly, while Treg cells (3.12% ± 0.13% vs 0.82% ± 0.05%) decreased in the combination therapy group, indicating remission of immunosuppression and enhanced immune response. Granzyme B secreted by CD8 cells in different groups was further evaluated by flow cytometry (Figures S20 and S21, Supporting Information). The level of granzyme B was highest in the combination therapy group and it could play the strongest role in inducing antitumor and immune responses. These results indicate that the combination therapy group produced more response molecules to trigger the immune response and enhance antitumor immunity, leading to synergistically enhanced antitumor immunity.

The HE‐stained images showed that there were obvious lung nodules in the PBS and anti‐PD‐L1 mAb groups, in which the black box represents lung nodules, indicating that anti‐PD‐L1 mAb had little effect on inhibiting tumor metastasis. However, no obvious lung nodules were observed in the ATO NPs@Au‐cRGD+UTMD+laser + anti‐PD‐L1 mAb group, verifying that the combination therapy could suppress in situ liver cancer lung metastasis (Figure 7H). Immunofluorescence was also used to evaluate angiogenesis in tumor tissues and platelet endothelial cell adhesion factor and the vascular marker CD31 were selected to evaluate angiogenesis. The PBS group showed more CD31 expression, indicating rapid tumor growth and metastasis. The ATO NPs@Au‐cRGD+UTMD+laser+anti‐PD‐L1 mAb group showed the lowest expression of CD31 (Figure 7I), which further confirmed that the combination therapy effectively inhibited tumor growth and metastasis by promoting tumor cell death. Collectively, our results indicated that our combination therapy can effectively slow the growth of primary liver tumors and a trigger systemic antitumor immune response to inhibit liver cancer lung metastasis.

## 3 Conclusion

This study reported the construction of a nano ultrasound contrast agent that could enable ultrasound imaging and stimulate immune responses by ferroptosis and PTT therapy more effectively than the free drug. Combined with the PD‐L1 Ab, the ATO/PFH NPs@Au‐cRGD nanodrug delivery system could effectively suppress liver orthotopic tumors and inhibit lung metastasis through activation of systemic immunity. These data support the concept of a combination of multiple treatments, lay the foundation for future research to address the clinical needs of liver cancer treatment, and may have implications for the treatment of other solid tumors beyond liver cancer.

## 4 Experimental Section

### Materials

ATO was purchased from Sinopharm Holding Co., Ltd. (Shanghai, China). PFH (MW:338.04) was purchased from Shanghai Macklin Biochemical Co., Ltd. (Shanghai, China). DSPE‐PEG2k‐SH were purchased from Ponsure Biotechnology Company (Shanghai, China). DOTAP (2,3‐Dioleoyloxy‐propyl‐trimethylammoniumchlorid) and DPPC (1,2‐dipalmitoyl‐sn‐glycero‐3‐phosphocholine) were purchased from CordenPharma Switzerland LLP (Switzerland). c(RGDfKC)‐SH (99.4% purity, cRGD, Mw = 706.83, sequence: cyclo[Arg‐Gly‐Asp‐(d‐Phe)‐Lys(Cys)]) was synthesized by ChinaPeptides Co., Ltd. (Shanghai, China). Poly(vinylalcohol) (PVA, 99% purity, Mw = 22 000 Da) were obtained from Sigma–Aldrich Co., Ltd. (Shanghai, China). All other chemicals and organic solvents were of analytical grade purchased from Sinopharm Chemical Reagent Co., Ltd. (Shanghai, China).

### Preparation of ATO/PFH NPs‐cRGD@Au

ATO/PFH NPs‐cRGD@Au were prepared according to methods described previously. First, 6 mg of DSPE‐PEG2k‐SH, 1.5 mg of DOTAP, and 1.5 mg of DPPC and 0.75 mg cholesterol were dissolved in 2 mL of trichloromethane. The solution was transferred into a round‐bottom flask. The organic solvent was removed by rotary evaporation at 55 °C under a vacuum (speed 120r /min) to form a uniform lipid membrane. Two milliliter phosphate‐buffered saline (PBS) was added to hydrate the lipid membrane. Then the emulsification was carried out by acoustic vibration instrument under the condition of ice bath. The acoustic vibration mode was set to pulse mode, the power was 400 W, and the time was 4 min. During the emulsification process, 50 µL PFH and 200 µL of ATO aqueous solution were added dropwise to obtain the liposomes. Then the liposome solution was centrifuged at 15 000 rpm for 15 min to remove the free lipid components and unencapsulated drugs to obtain the ATO/ PFH liposomes.

Gold nanoparticles (AuNPs) were decorated onto the liposome surface following a previous reported method with minor modification.^[^ ^40^ ^]^ Chloroauric acid (10 mm) was added and gently mixed with liposome suspension (1.5 mm lipid concentration) in a molar ratio of 1:4 until uniformly distributed, followed by the addition of the same volume of ascorbic acid (40 mm) solution. Following reduction, liposomes samples were purified by ultrafiltration to remove unreacted gold chloride and ascorbic acid. After decoration with AuNPs, the cRGD‐SH (500 µg) was added to the solution (5 mL) and mixed in the ice bath for 4 h. Finally, the solution was purified by ultrafiltration to obtain ATO/PFH NPs‐cRGD@Au and was stored at 4 °C. The preparations of other nanoparticles were obtained in the same manner with a slight revision.

### Characterization of ATO/PFH NPs‐cRGD@Au

The size distribution, zeta potential, and PDI of ATO/PFH NPs‐cRGD@Au were determined by Malvern Zetasizer Nano ZS (Malvern Instruments, Malvern, UK). TEM was performed by a Talos F200X transmission electron microscope (Shanghai, China) with a field emission gun operating at 200 kV. To assess the storage stability, ATO/PFH NPs‐cRGD@Au was stored at different temperatures (4, 25, and 37 °C) and observed for 7 days by dynamic light scattering (DLS) analysis. The ATO drug loading capacity was measured by an inductively coupled plasma mass spectrometry (ICP‐MS, Leeman Prodigy, USA). The drug loading efficiency (DL) and encapsulation efficiency (EE) were determined as described previously.^[^ ^57^ ^]^

### Drug Release of ATO/PFH NPs‐cRGD@Au

The release of ATO in ATO/PFH NPs‐cRGD@Au was detected by ICS‐MS at different pH values (6.5 and 7.4). Briefly, 2 mL of free ATO, and ATO NPs were put into a dialysis bag (Mw = 3500 Da) and sonicated. Next, the dialysis bags were placed in the 10 mL of phosphate‐buffered saline (PBS) solution with different pH. It was stirred at 100 rpm at 37 °C. The filtrate of different groups was collected at certain intervals and assessed by ICP‐MS. Each assay was repeated in triplicate. Cell lines and cell culture. The human hepatoma cell line Huh‐7 and mouse hepatoma cell line Hepa1‐6 were kindly provided by Stem Cell Bank, Chinese Academy of Sciences (Shanghai, China). Huh‐7 were cultured in Dulbecco's modified Eagle's medium (Gibco, Grand Island, USA) containing 10% fetal bovine serum (Gibco, Grand Island, USA) and 1% penicillin‐streptomycin solution (Gibco, Grand Island, USA) and 1% Glutamax (Gibco, Grand Island, USA) and 1% Sodium Pyruvate 100 mm Solution (Gibco, Grand Island, USA) in a humidified incubator with 5% CO_2_ at 37 °C. Hepa 1–6 were cultured in Dulbecco's modified Eagle's medium (Gibco, Grand Island, USA) containing 10% fetal bovine serum (Gibco, Grand Island, USA) and 1% penicillin‐streptomycin solution (Gibco, Grand Island, USA) and 1% Sodium Pyruvate 100 mm Solution (Gibco, Grand Island, USA) in a humidified incubator with 5% CO_2_ at 37 °C.

### In Vitro and In Vivo US Imaging

In vitro US imaging of ATO/PFH NPs‐cRGD@Au was carried out in a latex tube (with an inner diameter of 5 mm) using an Mindray Resona 7s ultrasound machine (Shenzhen Mindray Bio‐Medical Electronic Co, Shenzhen, China) equipped with a linear array transducer (3–11 MHz). The ATO/PFH NP@Au‐cRGD were dispersed in 0.9% saline and injected into the latex tube. Saline was used as the negative control. The US imaging was performed using the transducer in both conventional B mode ultrasound and contrast‐enhanced ultrasound (CEUS) (mechanical index, MI = 0.074) mode.

The in vivo US imaging of ATO/PFH NPs‐cRGD@Au was measured in tumor‐bearing nude mice using Mindray Resona 7s ultrasound machine (L14‐5 linear array transducer). The anesthetized tumor‐bearing nude mice were randomly divided into three groups (3 mice per group) and were separately injected intratumorally with 0.2 mL of saline, ATO/PFH NP@Au‐cRGD (4 mg mL^−1^) and Sonovue (SF_6_ = 4 mg mL^−1^). Saline was used as the negative control, and Sonovue was used as the positive control. Post‐injection images of tumors were captured by ultrasound machine.

### Ultrasound in vitro cell

A Sonicator740 therapeutic ultrasound apparatus from Mettler Electronics Crop (USA), with 1 MHz in frequency, 1, 2 and 3 W cm^−2^ in intensity, 30, 60, 90, and 120 s in exposure time, and 25 mm^2^ cross‐sectional area of the probe was applied. The transducer was placed under the cell culture plates and then 1–3 cm thick couplants were coated to form a conductive pathway of ultrasound waves. Finally, the cell culture plate was put onto the surface of the transducer.

### Cell uptake

The Huh‐7 and Hepa 1–6 cells were seeded in a 6‐well culture plate at a density of 1×10^4^ cells per well overnight and then exposed to Rhodamine B (RB) (10 µg mL^−1^), RB NPs@Au (10 µg mL^−1^), RB NP@Au‐cRGD (10 µg mL^−1^), and RB NP@Au‐cRGD +UTMD (10 µg mL^−1^). After they were incubated for 1 and 4 h, the cells were washed three times with PBS (pH 7.4), and the nucleus was labeled with Hoechst 33 342 (5 µg mL^−1^) and imaged using confocal laser scanning microscopy (FV1000, Olympus, Japan). To prepare the samples for flow cytometry, the Huh‐7 and Hepa 1–6 cells were seeded in a six‐well culture plate at a density of 2 × 10^5^ cells per well overnight and incubated with 10 µg mL^−1^ nanoparticles at different formulations for 1 and 4 h. The blank culture medium was treated as control. After incubation, the cells were washed three times with PBS. Then, the cells were detached with trypsin, washed three times with PBS, resuspended in PBS, and analyzed by flow cytometry (Becton Dickinson, USA).

### Cytotoxicity Assays

Cytotoxicity toward the Huh‐7 and Hepa1‐6 cells was determined quantitatively in vitro by the cell counting kit‐8 (CCK‐8) assay. Huh‐7 and Hepa1−6 cells were distributed in a 96‐well culture plate (5000 cells per well) and incubated overnight. Then, the original medium was discarded and different drug formulations were added in different concentrations for different incubation times. The blank culture medium was treated as control. After 4 h coincubation, the cells were irradiated with a 760 nm laser (1 W cm^−2^, 4 min) while the other groups were placed in the dark. The cells were treated with ultrasound at 1 MHz, 2.0 W cm^−2^ for 60 s. Then, the cells were further incubated for 12 h. Subsequently, cytotoxicity was evaluated by CCK‐8 assay (Biosharp, Beijing, China) according to the manufacturer's instructions. Cell viability was detected by the standard CCK‐8 protocol. Each assay was repeated in triplicate.

### ROS Determination

The total induction of ROS in the Huh‐7 and Hepa 1–6 cells caused by different drugs was determined by fluorescence microscopy and flow cytometry. The Huh‐7 and Hepa1‐6 cells were seeded in a six‐well culture plate at a density of 2 × 10^4^ cells per well overnight and treated with AuNPs, ATO, ATO NPs@Au, ATO NPs@Au ‐cRGD, ATO NPs‐cRGD@Au +UTMD, ATO NPs@Au‐cRGD +UTMD+laser at an equivalent concentration of 15 or 2 µm for 12 h. Nontreated tumor cells were used as control. The supernatants were removed, and dichlorofluorescein diacetate (DCFH‐DA, 5 mg mL^−1^) in DMEM was added. After incubating at 37 °C for 30 min, the cells were washed three times with PBS. Then, the cells were incubated with 4% paraformaldehyde (1 mL per dish) for 10 min. The cells were washed with PBS three times and then observed on a fluorescence microscope (CFM‐500, Zeiss, Germany). To prepare the samples for flow cytometry, the Huh‐7 and Hepa1‐6 cells were seeded in a six‐well culture plate at a density of 2 × 10^5^ cells per well overnight and incubated with different formulations of nanoparticles at 15 or 2 µm for 12 h. The blank culture medium was treated as control. The supernatants were removed, and DCFH‐DA (5 mg mL^−1^) in DMEM was added. After incubating for 30 min, the cells were washed three times with PBS. Then, the cells were detached with trypsin, washed three times with PBS, resuspended in PBS, and analyzed by flow cytometry (Becton Dickinson, USA).

### Intracellular Lipid Peroxide Measurement

A BODIPY 581/591 C11probe (Thermo Fisher Scientific, Shanghai, China) was used to investigate the production of LPO in PBS, AuNPs, ATO, ATO NPs@Au, ATO NPs@Au‐cRGD, ATO NPs@Au‐cRGD+UTMD, ATO NPs@Au‐cRGD+UTMD+Laser treated Huh7 and Hepa1‐6 cells. Briefly, 2 × 10^5^ cells per well were seeded in the 6‐well culture plate. After reaching 70–80% confluence, cells were incubated with different formulations for 6 h. After removing the medium, cells were washed with PBS for three times, and stained with C11‐BODIPY (10 µm, in free medium) for 20 min. The cells were incubated with 4% paraformaldehyde (1 mL per dish) for 10 min after washing with PBS. The cells were washed with PBS three times and then observed on a fluorescence microscope (CFM‐500, Zeiss, Germany). Similarly, after washing with PBS for three times, cells were collected and subjected to flow cytometry.

### Western Blotting

The western blotting was performed according to standard procedures. The primary antibody against GPX4, xCT, and GAPDH were obtained from Abcam plc (Cambridge, MA, UK). All the secondary antibodies were obtained from Cell Signaling Technology (Danvers, MA, USA). Each assay was repeated in triplicate.

### Evaluation of the Repolarization of M1 Macrophages

THP‐1 cells were seeded in 24‐well culture system with 0.4 µm polycarbonate porous membranes, and IL‐4 (20 ng/mL) was added to stimulate THP1 cells to differentiate into M2 macrophages. Then, the Huh7 cells were co‐incubated with PBS, AuNPs, ATO, ATO NPs@Au, ATO NPs@Au ‐cRGD, ATO NPs@Au ‐cRGD + UTMD, ATO NPs@Au ‐cRGD + UTMD+Laser. After that, the cells were collected and labeled by using the following anti‐human antibodies: CD11b‐FITC (#101206, BioLegend), CD86‐BV605 (#305430, BioLegend), CD206‐BV421 (#321125, BioLegend). After PBS washing, the cells were analyzed via flow cytometry.

### CRT Expression Assays

Huh7 cells (8 × 10^5^) and Hepa1‐6 cells were seeded in six‐well plate overnight, then treated with PBS, AuNPs, ATO, ATO NPs@Au, ATO NPs@Au‐cRGD, ATO NPs@Au‐cRGD + UTMD, ATO NPs@Au‐cRGD+UTMD+laser and incubated for 24 h. Subsequently, cells washed with cold PBS. After PBS washing, cells were collected and incubated with an Alexa Fluor 647‐conjugated anti‐CRT antibody (ab196159, 1/50 dilution, Abcam) for 30 min and analyzed via flow cytometry.

### In Vitro ATP and HMGB1 Extracellular Release Assays

Enhanced ATP Assay Kit (Beyotime, Shanghai, China) and HMGB1 enzyme‐linked immunosorbent assay kits (Shanghai Enzyme‐linked Biotechnology) were used to detect the extracellular release of ATP and HMGB1, respectively. Huh7 cells and Hepa1−6 cells (8 × 10^5^ cells per well) were seeded in a six‐well plate overnight. Next, cells were treated with PBS, AuNPs, ATO, ATO NPs@Au, ATO NPs@Au‐cRGD, ATO NPs@Au‐cRGD+UTMD, ATO NPs@Au‐cRGD+ UTMD+laser for 24 h. Then, the supernatant was collected and tested under the guidance of manufacturer's instructions.

### Experimental Animals

Male 4−6‐week‐old BALB/c and C57BL/ 6N mice were obtained from Zhongshan Hospital and raised in the SPF animal facility. All the animal experimental procedures were performed according to the protocols approved by Ethics Institute of Zhongshan Hospital (2022‐003).

### Pharmacokinetic Study

SD rats were used for pharmacokinetic studies of the ATO. In brief, SD rats were randomly divided into three groups (n = 3), and ATO formulations were delivered by tail vein injection at a dose of 1 mg kg^−1^. Blood samples of rats were collected at the desired times (0.25, 0.5, 1, 2, 4, 8, 24, 36, 48, 60, 72 h). Then, the samples were centrifuged and stored at −80 °C for ICP‐MS analysis. The pharmacokinetic parameters were analyzed by Phoenix WinNonlin (version 8.1).

### In Vivo Distribution

Free Dir, Dir NPs, and Dir NPs‐RGD of 200 µL (Dir 10 µg mL^−1^ equiv) was injected into the mice via the tail vein when the tumor volume reached ≈200 mm^3^. Fluorescence images were analyzed with an in vivo imaging system (Berthold Technologies, Germany) at 2, 4, 8, 24, and 48 h. Then, the mice were sacrificed after 48 h, and the organs were collected to measure the fluorescence intensity. In addition, ATO, ATO NPs@Au, and ATO NPs@Au‐cRGD were injected into the mice via the tail vein when the tumor volume reached ≈200 mm^3^. The concentrations of ATO were 1 mg kg^−1^. After 24 h of the administration, mice were sacrificed, and the main organs were collected to measure the drug distribution by ICP‐MS.

### Antitumor Efficiency

The BALB/c subcutaneous transplantation tumor models were established by injecting the flanks of the mice with 5 × 10^6^ Huh7. BALB/c mice bearing 100 mm^3^ tumors were randomly divided into seven treatment groups: PBS, AuNPs, ATO, ATO NPs@Au, ATO NPs@Au‐cRGD, ATO NPs‐cRGD@Au+UTMD, ATO NPs@Au‐cRGD+UTMD+Laser (ultrasound was implemented at 1 MHz and 2 W cm^−2^ for 60 s and the laser was irradiated with a 760 nm laser at 1 W cm^−2^ for 4 min). The dose of ATO was 1 mg kg^−1^ and the concentration of various group was applied for subsequent animal experiments except for the PBS and AuNPs group. The mice were injected via the tail vein every three days, and the weight and volume of the mice were measured and recorded every 4 days. The volume of the tumor was determined according to the equation *V* = *ab* ^2^ × 1/2, where *a* and *b* were the longest and shortest diameters of the tumors. After 16 days of treatment, the tumors and organs were harvested, weighed, and imaged after the mice were euthanized. The homogenates were centrifuged, and the levels of the IFN‐*γ*, and TNF‐*α* (ELISA, Invitrogen) in the supernatant were respectively measured using an ELISA kit under the guidance of manufacturer's instructions. Next, the tumors and organs were washed with PBS three times, fixed with 10% neutral buffered formalin solution, embedded in paraffin, and cut into sections. H&E, TUNEL, Ki67, and staining were used to histopathologically analyze the tumor sections under a light microscope. Additionally, tumor immunohistochemistry was applied to assess the expression of GPX4. Finally, H&E staining was also conducted for various important organs, including the heart, liver, spleen, lung, and kidney.

### Orthotopic Liver Tumor Experiment

The C57BL/6N subcutaneous transplantation tumor models were established by injecting the flanks of the mice with 5 × 10^6^ Hepa1−6 cells. Then, the mice were anesthetized with 1% pentobarbital (50 mg kg^−1^, intraperitoneal injection) and the tumors excised and cut into pieces. Similar‐sized tumoroids were transplanted into the liver of new C57BL/6N mice to establish the orthotopic xenograft liver tumor models. One week after the surgery, the mice were imaged by ultrasound and grouped based on size of the liver tumor. Tumor growth was measured by ultrasound (10L‐4 linear array transducer (Siemens Acuson Sequoia) at days 0, 8, and 16. After the treatment, the tumors and lungs were harvested. The IL‐12, IFN‐*γ*, and TNF‐*α* (ELISA, Invitrogen) of the tumors were respectively measured using an ELISA kit. Western blot of the orthotopic liver tumor was performed to evaluate the protein level of xCT. Lung tissue HE and tumor CD31 staining were used to evaluate lung metastasis and tumor invasion of orthotopic liver tumor in situ under different interventions.

### In Vivo Immune Stimulation Experiment

As described above, tumors and LNs were separated from different mice groups. The samples of LNs were prepared for flow cytometry to detect the maturation of DCs. Then, the scissor was used to cut the tumor, making the tumor fragments as small as possible. These fragments are digested through digestive liquid. This liquid was filtered and transferred to another centrifugal tube and centrifuged at 400 × *g* at 4 °C for 5 min. Then, the liquid was gradient centrifuged by using different concentrations of isotonic Percoll solution. A Pasteur tube was used to extract the cell population in the middle of the liquid, and the cells were centrifuged at 400 × *g* at 4 °C for 5 min. After centrifugation, the red blood cell (RBC) lysate was added to lyse RBCs and the remaining cells were cleaned and stained with different immunofluorescent antibodies: Fixable Viability Stain 780 (#565388, BD Biosciences), CD3*ε*‐PE (#100307, BioLegend), CD4‐FITC (#100405, BioLegend), CD8a‐ PerCP/Cyanine5.5 (#100734, BioLegend), CD11c‐PE (# 117307, BioLegend), CD80‐APC (#104714, BioLegend), CD86‐PE/Cyanine7 (#105014, BioLegend), CD25‐APC(#101910, BioLegend), Granzyme B—APC (#372204, BioLegend). CD11c‐PE antibodies, CD80‐APC antibodies, and CD86‐PE/Cyanine7 antibodies were used to represent the maturation of DCs. CD3*ε*‐PE antibodies, CD4‐FITC antibodies, and CD25‐APC antibodies were used on behalf of Treg cells, and the cells stained with CD3*ε*‐PE antibodies and CD4‐FITC antibodies or CD8a‐PerCP/Cyanine5.5 antibodies were separately CD4^+^ T cells or CD8^+^ Tcells, respectively. CD3*ε*‐PE antibodies, CD8a‐PerCP/Cyanine5.5 antibodies, and Granzyme B—APC antibodies were Cytotoxic T lymphocytes (CTLs).

### Statistics

The data were presented as mean ± SD values. Statistical analyses were performed by either unpaired Student's *t*‐test or one way‐analysis of variance using GraphPad Prism 8 software. The survival curves were created using the Kaplan–Meier method. The statistical significance of differences was set at *p* < 0.05.

## Conflict of Interest

The authors declare no conflict of interest.

## Supporting information

Supporting Information

## Data Availability

The data that support the findings of this study are available on request from the corresponding author. The data are not publicly available due to privacy or ethical restrictions.
