# Supplementary material for: Nano Ultrasound Contrast Agent for Synergistic Chemo‐photothermal Therapy and Enhanced Immunotherapy Against Liver Cancer and Metastasis
Source: Adv Sci (Weinh). 2023 May 10;10(21):2300878. doi: 10.1002/advs.202300878 (PMC10375134; doi:10.1002/advs.202300878)
Supplement: Supplementary file 1 — Supporting Information [file ADVS-10-2300878-s001.pdf]

ADVANCED SCIENCE
Open Access

Supporting Information

for *Adv. Sci.*, DOI 10.1002/advs.202300878

Nano Ultrasound Contrast Agent for Synergistic Chemo-photothermal Therapy and Enhanced Immunotherapy Against Liver Cancer and Metastasis

Yijie Qiu, Zhihua Wu, Yanling Chen, Jinghan Liao, Qi Zhang, Quan Wang, Yi Duan, Ke Gong, Sheng Chen, Liting Wang, Peili Fan, Yourong Duan, Wenping Wang* and Yi Dong**

Supporting information

Nano Ultrasound Contrast Agent for Synergistic Chemo-photothermal Therapy and Enhanced Immunotherapy Against Liver Cancer and Metastasis

Authors

Yijie Qiu[a,1], Zhihua Wu[b,1], Yanling Chen[a], Jinghan Liao[b], Qi Zhang[a], Quan Wang[b], Yi Duan[b], Ke Gong[b], Sheng Chen[a], Liting Wang[b], Peili Fan[a], Yourong Duan[b,*], Wenping Wang[a,*], Yi Dong[c,*]

This file includes: Figure. S1 to S21, Table. S1

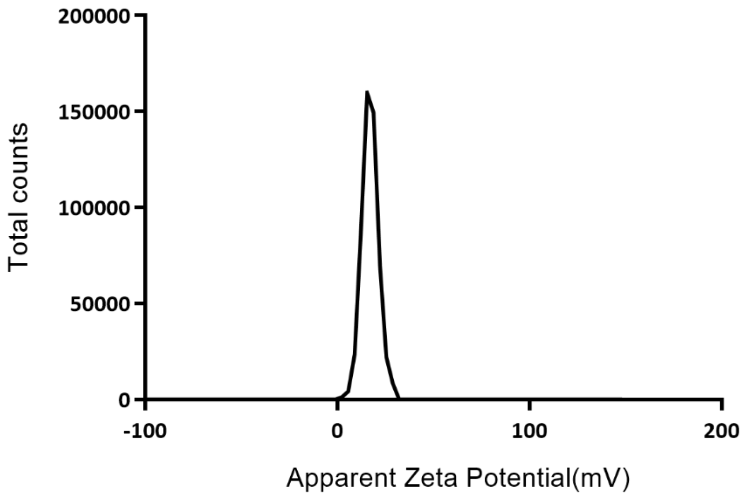

Figure S1 The zeta potential of ATO/PFH NPs@Au-cRGD was 10.47 ± 1.20 mV.

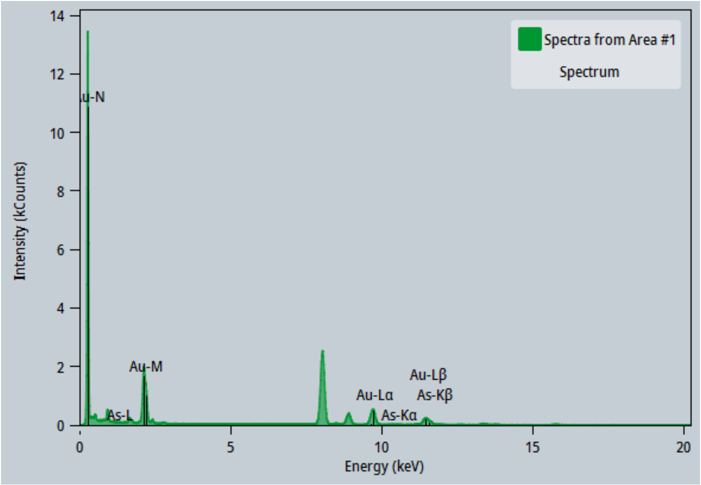

Figure S2 EDS spectrum of liposomes. Au peaks suggesting the presence of AuNPs on the liposome surface.

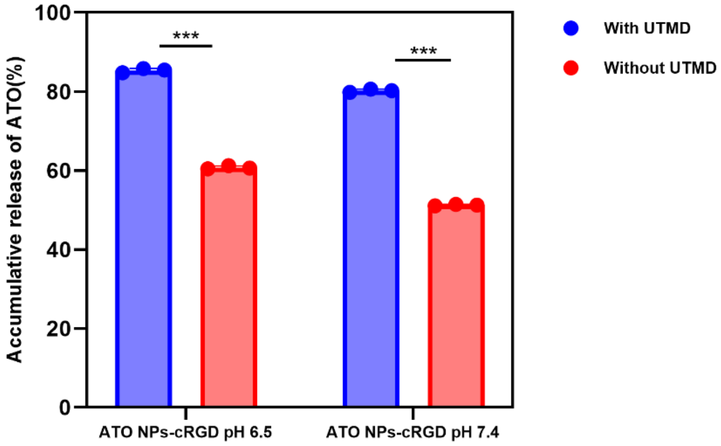

Figure S3. In vitro release of ATO from ATO NPs@Au-cRGD with or without UTMD treatment at pH 6.5 and pH 7.4. ***$p < 0.001$.

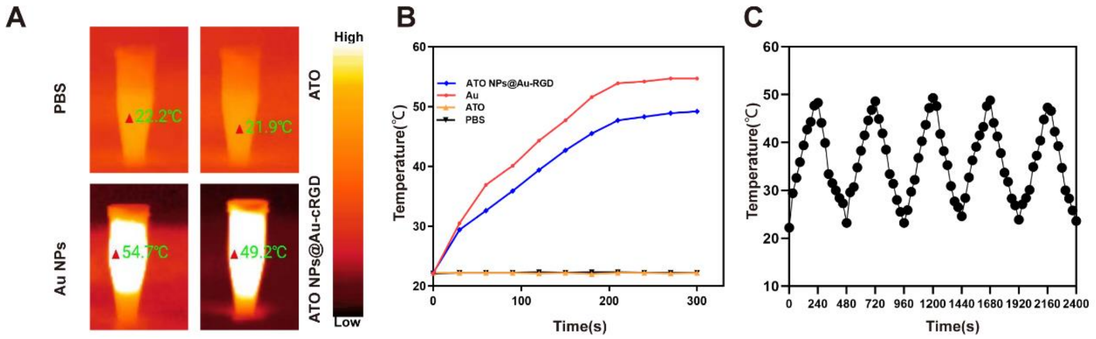

Figure S4 Representative thermal images (A) and photothermal effects (B) of ATO NPs@Au-RGD, ATO, AuNPs and PBS. (C) Photothermal effects of ATO NPs@Au-cRGD (under NIR irradiation (1.0 W/cm^2) after five irradiation-cooling cycles.

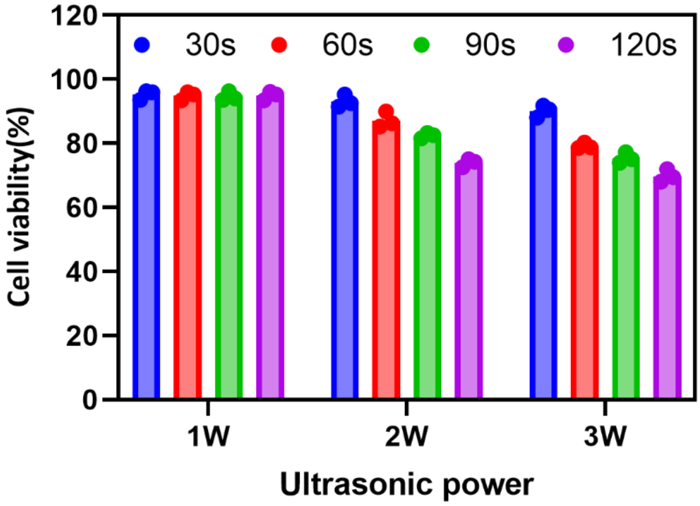

Figure S5 Huh7 cell viability after treated with various power and time of ultrasound therapeutic apparatus via CCK8 assay.

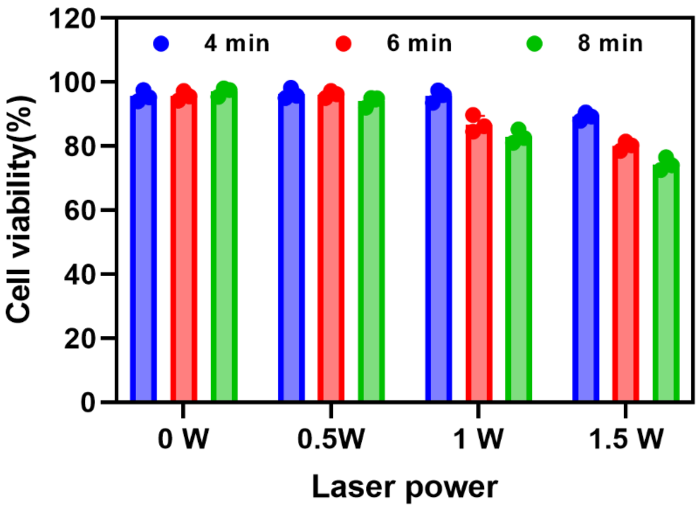

Figure S6 Huh7 cell viability after treated with various power and time of NIR laser apparatus via CCK8 assay.

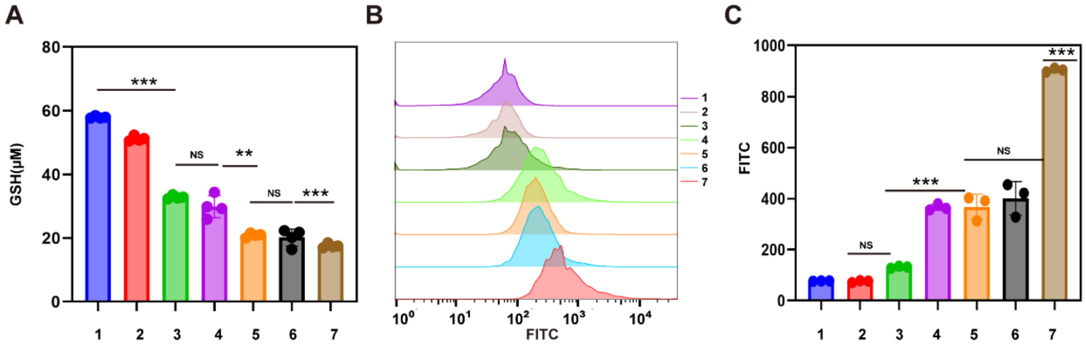

Figure S7 Mechanism of increased ROS and GSH depletion induced by ATO/PFH NPs@Au -cRGD. (A) GSH levels in Hepa1-6 cells treated with different interventions. (B) Flow cytometry analysis of ROS in Hepa1-6 cells with different treatments. (C) Quantification of flow cytometry analysis of Hepa1-6 cells with different treatments. (1, PBS; 2, AuNPs 3, Free ATO; 4, ATO NPs; 5, ATO NPs-cRGD; 6, ATO NPs-cRGD+UTMD; 7, ATO NPs-cRGD+UTMD+laser). All data are presented as the mean ± SD, *$p < 0.05$, **$p < 0.01$, ***$p < 0.001$.

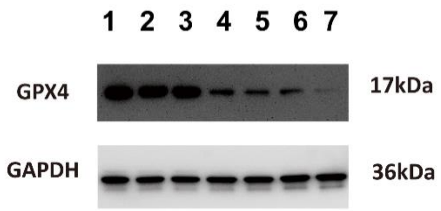

Figure S8 Western blotting results of GPX4 expression in Hepa1-6 cells after different treatment. (1, PBS; 2, AuNPs 3, Free ATO; 4, ATO NPs; 5, ATO NPs-cRGD; 6, ATO NPs-cRGD +UTMD; 7, ATO NPs-cRGD+UTMD+laser).

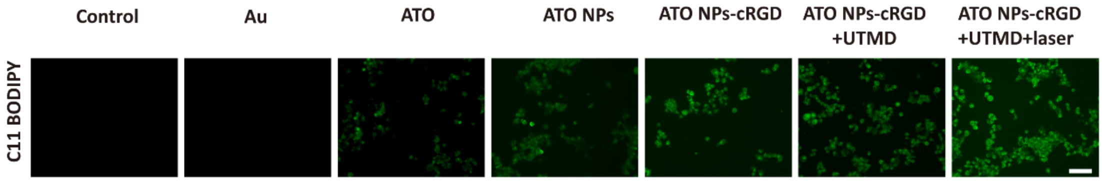

Figure S9 Fluorescence images of LPOs in Hepa1-6 cells. scale bar=100 μm.

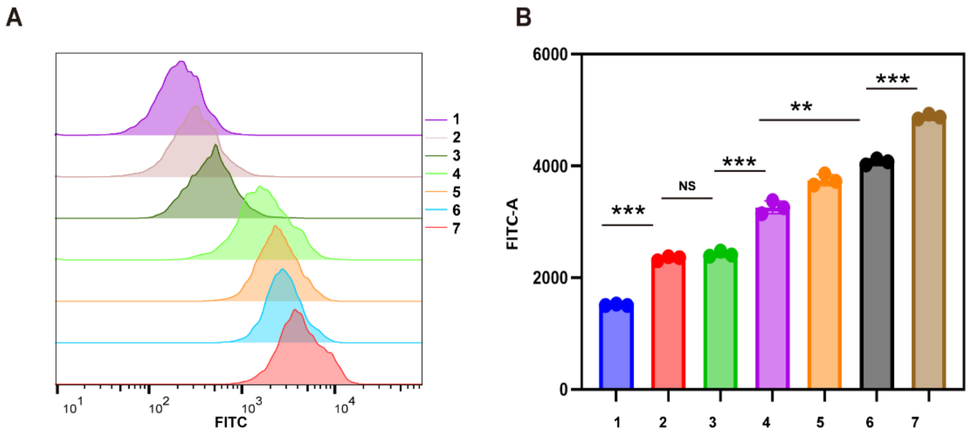

Figure S10 Flow cytometry analysis (A) and quantitative fluorescence intensity of LPOs (B) in Hepa1-6 cells with different treatments. (1, PBS; 2, AuNPs 3, Free ATO; 4, ATO NPs; 5, ATO NPs-cRGD; 6, ATO NPs-cRGD +UTMD; 7, ATO NPs-cRGD +UTMD+laser). All data are presented as the mean ± SD, $**p < 0.01$, $***p < 0.001$.

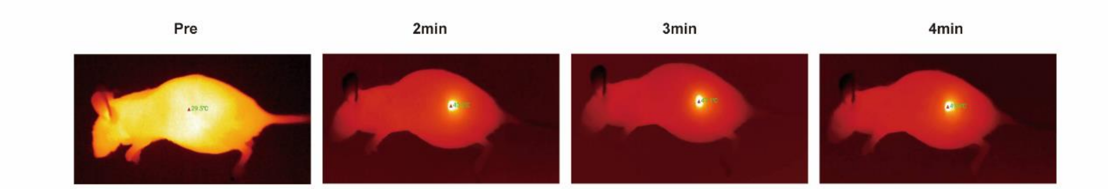

Figure S11 (A) Infrared thermal images of Huh7 tumor-bearing mice in the ATO NPs@Au-cRGD+UTMD+laser groups at different time points.

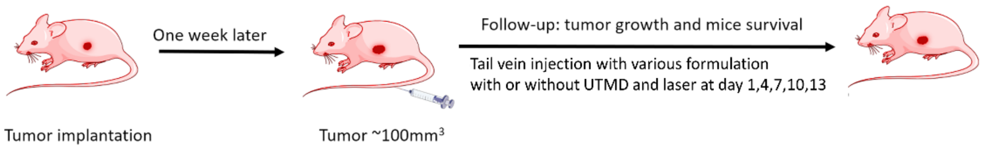

Figure S12 Treatment schedule of subcutaneous tumor in BALB/c mice with different formulation injections.

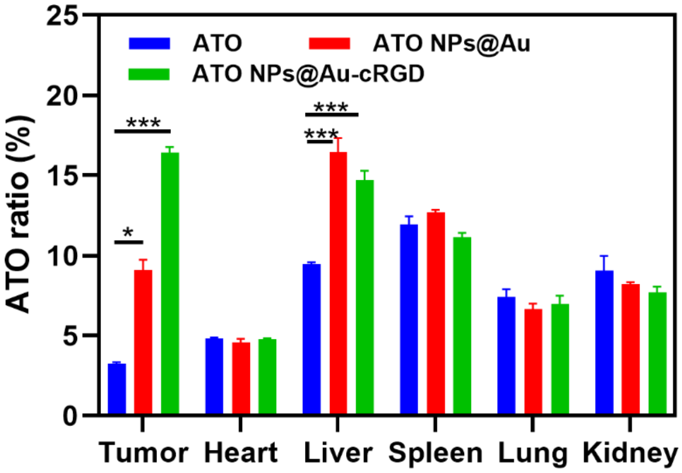

Figure S13 Quantified ATO concentration in major organs from ATO, ATO NPs@Au, and ATO NPs@Au-cRGD treated mice (n = 3) at 24h after intravenous injection. *p <0.05, ***p < 0.001.

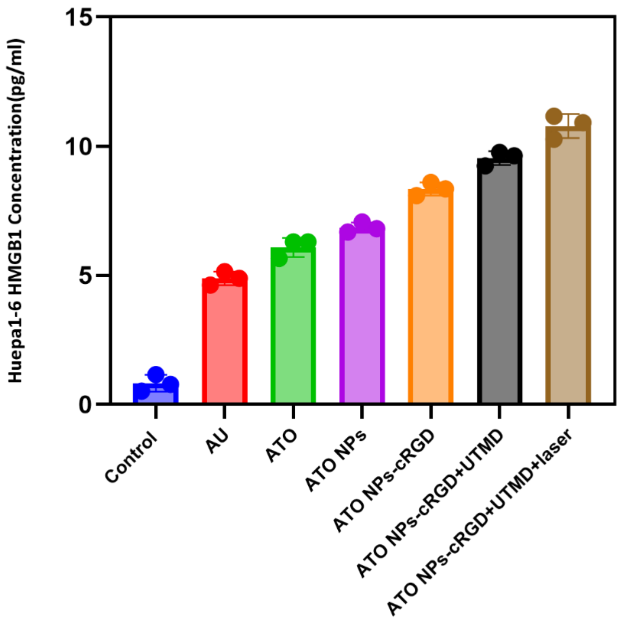

Figure S14 HMGB1 released from Hepa1-6 cells detected by an enzyme-linked immunosorbent assay (ELISA) kit.

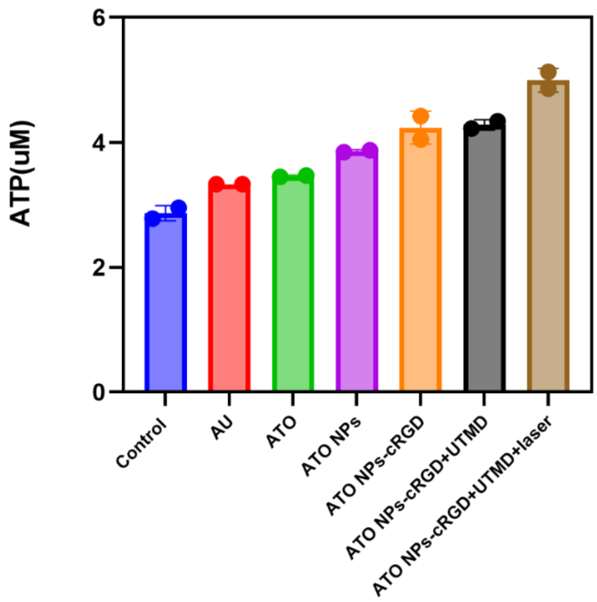

Figure S15 ATP secretion of Hepa1-6 cells detected by an enhanced ATP assay kit

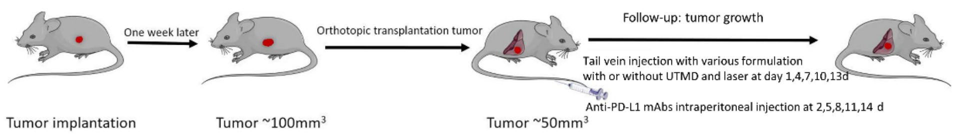

Figure S16 Treatment schedule of orthotopic liver tumor in C57BL/6N mice with different formulation injections.

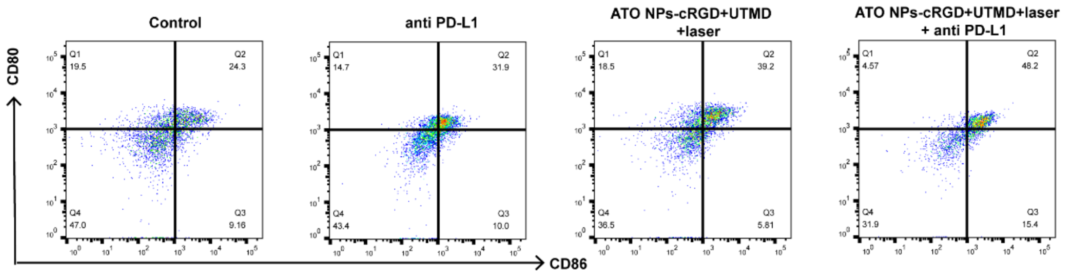

Figure S17 Flow cytometric data of CD11c+CD80+CD86 T cells in different groups.

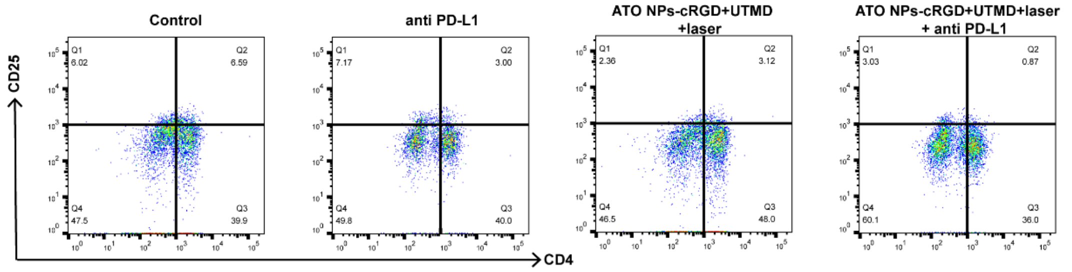

Figure S18 Flow cytometric data of CD4+CD25+ T cells in different groups.

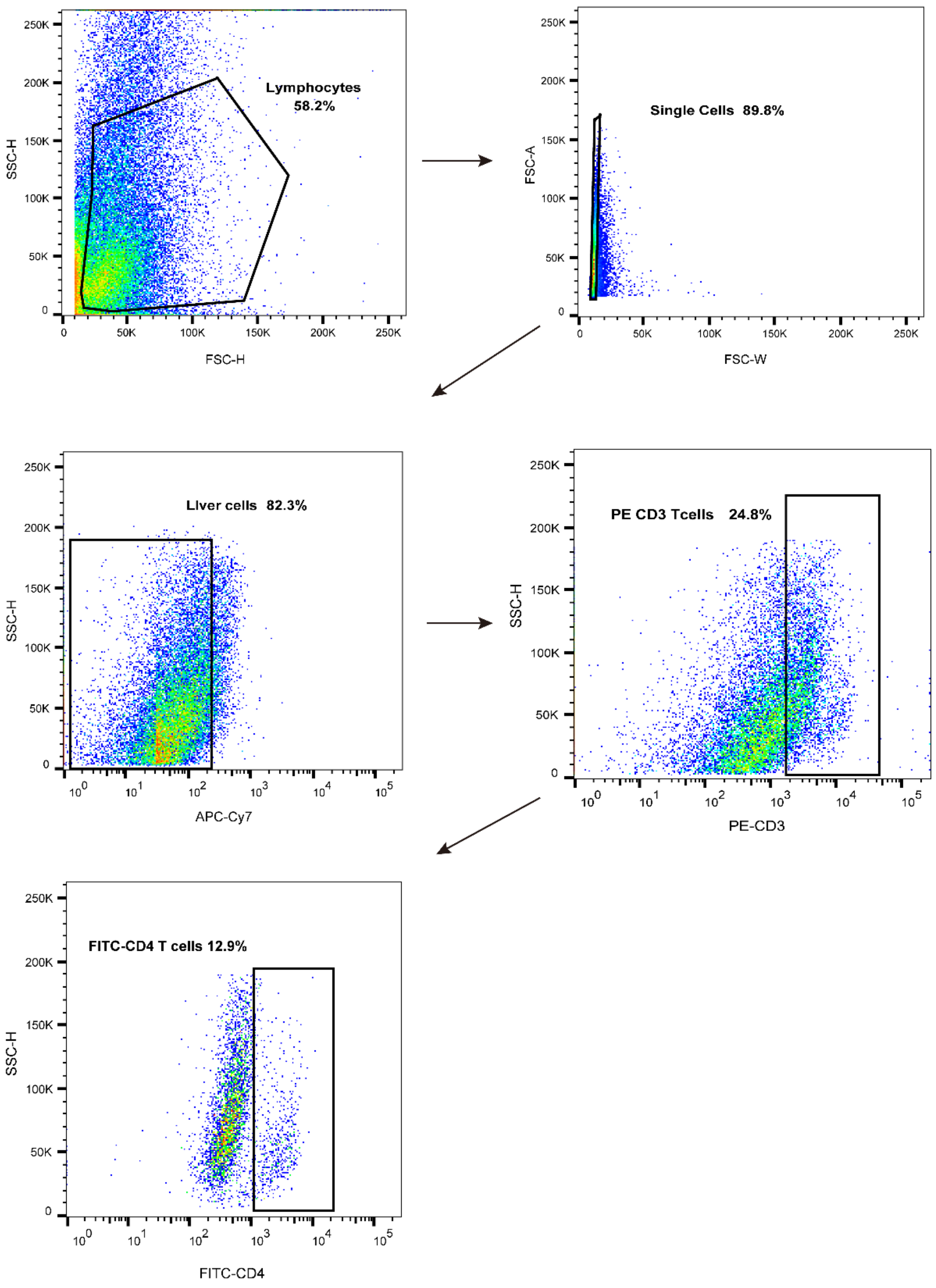

Figure S19 The gating strategy of CD4+ T cells in tumor from sacrificed mice.

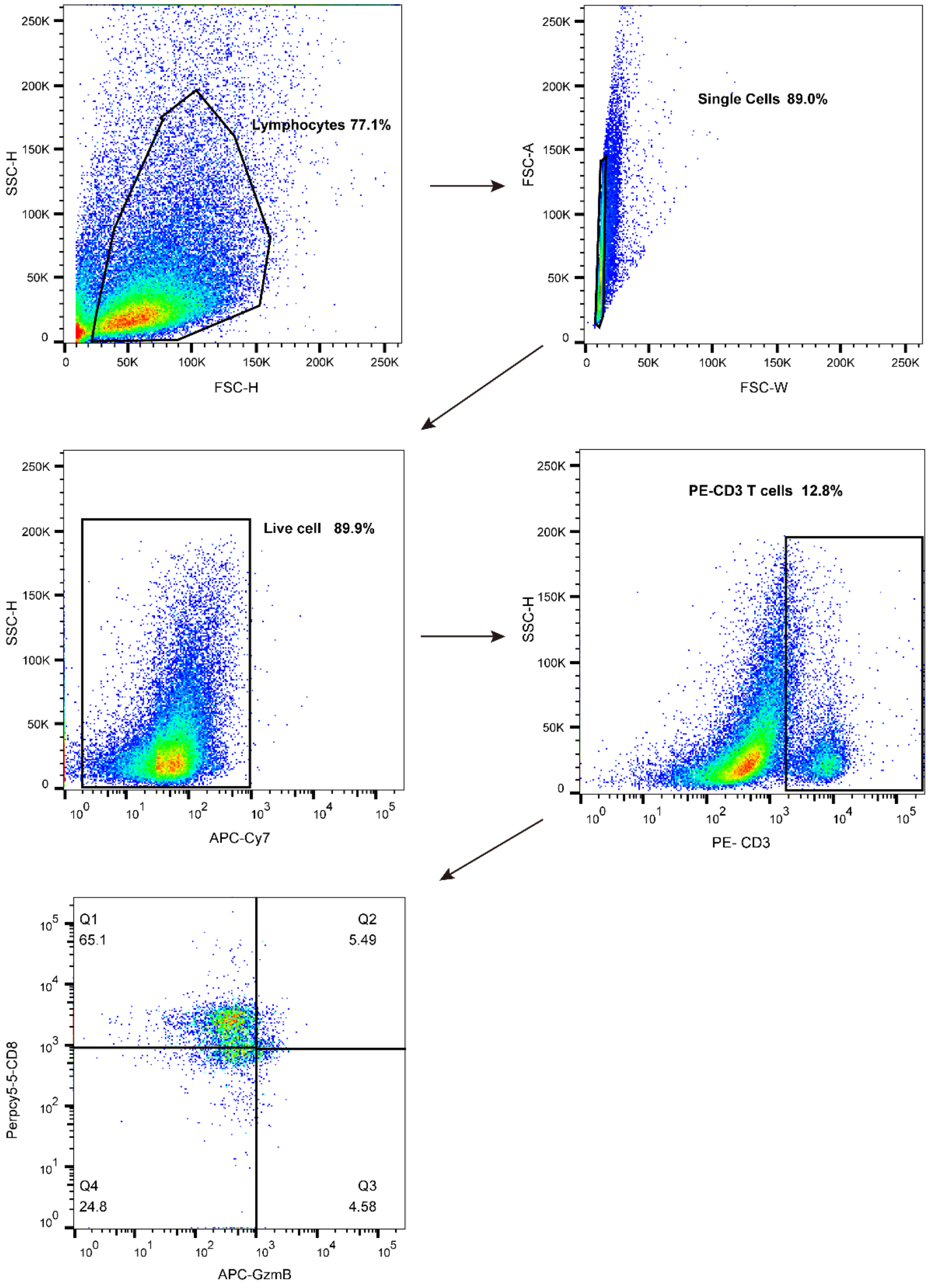

Figure S20 The gating strategy of Granzyme B secreted by CD8 T cell tumor from sacrificed mice.

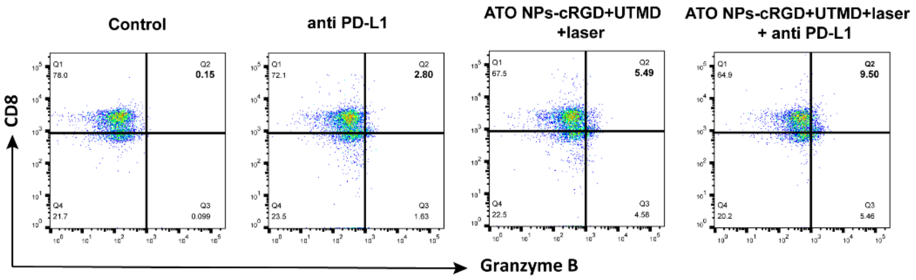

Figure S21 Flow cytometric data of Granzyme B in different groups.

Table

Table S1 Main pharmacokinetic parameters of ATO after vein injection in rats (n = 3).

Parameters	ATO	ATO NPs @ Au	ATO NPs @ Au-cRGD
Half-life (h)	19.45±0.81	39.56±3.78**	43.34±2.24***
CL (mL/h/kg)	160.51±9.54	46.36±2.21***	44.30±0.64***
AUC_{0-t} (μg x h/mL)	5.72±0.33	13.15±0.43***	15.32±0.17***
MRT (h)	19.51±0.05	27.49±0.36***	27.64±0.26***

** $P < 0.01$, *** $P < 0.001$ vs ATO group
